# Socio-emotional strengths against psychopathology and suicidal ideation in fear of COVID-19

**DOI:** 10.1007/s12144-021-02185-6

**Published:** 2021-09-28

**Authors:** Victoria Soto-Sanz, Raquel Falcó, Juan C. Marzo, José A. Piqueras, PSICO-RECURSOS COVID-19 study group, Alfonso Lopez-Nuñez, Agustín E. Martínez-González, Ornela Mateu, Beatriz Moreno-Amador, David Pineda, Maria Rivera-Riquelme, Tíscar Rodríguez-Jiménez, Verónica Vidal-Arenas

**Affiliations:** 1 Department of Health Psychology, Faculty of Social and Health Sciences, University Miguel Hernandez de Elche, Avda. de la Universidad, s/n. Edf. Altamira, 03202 Elche, Alicante, Spain; 2 Center for Applied Psychology, Miguel Hernandez University (UMH), Elche, Spain; 3 University of Alicante, Alicante, Spain; 4 Catholic University of Murcia, Murcia, Spain; 5 Jaume I University (UJI), Castellón de la Plana, Spain

**Keywords:** Suicidal ideation, Anxious-depressive symptoms, Covitality, Fear of COVID-19

## Abstract

Coronavirus disease (COVID-19) has caused a global health crisis. It also leads to different types of psychosocial problems in society as a result of preventive health measures and the disease itself. Among others, psychopathological symptoms and suicide behaviors have increased. The PsicorecurSOS COVID-19 online protocol was designed. At baseline, 1020 Spanish adults were assessed, during confinement, for sociodemographics, fear of COVID-19, anxious-depressive symptoms, covitality, and suicidal ideation. Reliability, descriptive, and frequency analyses were carried out, and the computer tool SPSS PROCESS was used to carry out a conditional process analysis (model 59). A total of 595 participants were included (58.30% response rate from baseline; mean age = 37.18 [*SD* = 13.30]; 72.44% female). Regarding suicidal ideation, 12% responded differently to “never,” 19.3% exceeded the cutoff point on the anxiety scale, and 24% on the depression scale. Moderate mediation analysis explained 27% of the variance in suicidal ideation. In addition, the indirect effect of moderate mediation was significant (b = −.004, *SE* = .002 with the presence of covitality; and b = .01, *SE* = .003 absence of covitality). Sex and age did not influence the overall outcome of the model. The data from this study can serve as a starting point for generating social and health treatment initiatives based on self-examination of anxiety-depressive symptoms and increasing socio-emotional skills in order to prevent and alleviate the psychosocial effects of the pandemic.

## Introduction

Since early 2020, coronavirus disease (COVID-19) has triggered a global health crisis, and in April, the World Health Organization (WHO) declared it a pandemic (WHO, 2020). It is also leading to different types of psychosocial problems in society as a result of preventive health measures and the disease itself.

During the first months of the pandemic, Spain was one of the countries most affected in terms of the number of people affected and deaths. Due to this situation, the government decreed a “state of alarm” by adopting forced confinement measures on March 14 (Ministry of the Presidency, Relations with the Courts and Democratic Memory, 2020), which lasted until the beginning of May, when a four-phase de-escalation began, which lasted until June 21, when the whole country returned to the so-called “new normality.” This new normality meant the end of most restrictions on mobility, seating capacity, etc., with the exception of the measures included in the laws and regulations defining the new normality, such as the use of masks in public places and the promotion of teleworking as well as better early detection in retirement homes.

Changes can occur in one’s psychological status during health emergencies, which can have consequences at the emotional and cognitive levels (Acherman et al., 2009; Mortensen, Becker, Ackerman, Neuberg & Kenrick, 2010; Schaller & Murray, 2008). Although the current pandemic situation is unprecedented, some studies have already reported on the psychological impact that preventive measures being implemented produce in the general population—specifically, forced quarantine.

One of these psychological impacts translates into fear. Fear, understood as an emotionally unpleasant state triggered by the perception of threatening stimuli (de Hoog et al., 2008), is a relevant factor in the pandemic. In this type of health crisis, people may have an excessive fear of the disease or related factors. This fear has been associated with suicide (Dsouza, Quadros, Hyderabdwala, & Mamun, 2020; Goyal, Chauhan, Chhikara, Gupta, & Singh, 2020; Mamun & Griffiths, 2020) and symptoms of anxiety and depression (e.g., Fitzpatrick, Harri,s & Dawve, 2020; Harper, Satchell, Fido, & Latzman, 2020; Satici et al., 2020b). Regarding the presence of anxiety and depression symptoms in Spanish population, recently, a study by González-Sanguino et al. (2020) has provided information about the first three weeks of the state of alarm with a sample of 3480 Spanish adults; the scores on the depression scale averaged 1.60 (*SD* = 1.50) with 18.7% of the sample exceeding the cut-off point on the scale PHQ-2 for detecting a possible case of depressive disorder. In anxiety, reported mean scores of 1.79 (*SD* = 1.63), with 21.6% of the sample exceeding the cut-off point on the PCl-C-2.

This increase in anxious-depressive symptoms can cause serious repercussions. In previous studies, the occurrence of anxiety and depressive disorders, or the presence of symptoms, has been associated with suicidal behavior (Gili et al., 2019; Gilmour, 2016; Sareen et al., 2005; Soto-Sanz et al., 2019). In relation with suicidal behavior during the pandemic of COVID-19, some studies reflect the impact of the pandemic on this health problem. For example, Caballero-Dominguez, Jimenez-Villamizar, & Campo-Arias(2020), where a sample of 700 Colombian responded to the four items CES-D-IS and found that 7.6% of the participants at a high suicide risk, where within a total score of 12.9 was considered for the cutoff score. There is also information on suicide behavior in the U.S. sample. For example, in a conducted through Qualtrics Panels using quota sampling methods with a general population sample of 10,625 people reported that since March 18 to April 4, 4.6% and 1.2% past-month suicidal ideation and suicide attempts, respectively, and since April 2 to 8, 10.7% seriously considered suicide in past 30 days, among 5412 subjects (Czeisler, et al., 2020). However, no studies on prevalence during this period in the Spanish population have been found in the scientific literature.

It is important to note that the COVID-19 pandemic has not only had an impact on emotional distress, but has also affected psychological well-being. Likewise, concerning the psychological states of people in confinement, a study of 17,000 users of a Chinese social network found that messages about depression and anxiety increased, and those expressing positive emotions decreased (Li, Wang, Xue, Zhao, & Zhu, 2020). Emotional well-being in relation to mental health problems is crucial because it is related to positive adaptation and coping, which, in turn, reduces risk factors for such problems (Dienel & Tay, 2015; Weare, 2015). Regarding the protective factor of emotional expression and regulation, Fernandez-Berrocal(2020) reported a negative association between emotional regulation and psychological disorders’ symptomatology. In turn, it has been found that people with higher emotional intelligence (EI) scores, whether measured using the skill model (Mayer & Salovey, 1997) or mixed model (Bar-On, 1997; Goleman, 1995; Petrides, Pita, & Kokkinaki, 2007), present less suicidal behavior (Domínguez-García & Fernández-Berrocal, 2018; Korkmaz et al., 2020).

In the positive psychology framework, the Covitality model has recently emerged, which is understood as the “synergistic effect of positive mental health resulting from the interaction between multiple positive psychological elements” (Renshaw et al., 2014, p. 14). These elements include belief in self, belief in others, emotional competence, and engaged living (Furlong, You, Renshaw, O’Malley, & Rebelez, 2013). The role of Covitality is being studied in different countries among children, adolescents, youth, and adults.

Data analyzed in a study in Spain on young university students (1029 participants) reflected that the absence of Covitality explained 23% of the presence of internalizing symptoms (anxiety and depression) and externalizing symptoms (behavioral problems). In addition, covitality was shown to moderate the relationship between suicidal behaviors (ideation and attempt) and internalizing symptoms, explaining 15% of the variance in suicidal behaviors (Soto-Sanz et al., 2019).

Therefore, fear of COVID-19, symptoms of anxiety and depression, and aspects of well-being seem to be related. For example, Satici et al. (2020b) showed a statistically significant and negative relationship between fear of COVID-19 and psychological well-being. Thus, COVID-19 and confinement increase fear, and fear can lead to symptoms of anxiety and depression and, consequently, suicidal ideation. However, the protective role of psychological strength is unknown, as some people with fear of COVID-19 and anxious-depressive symptoms develop ideation, while others do not.

In short, in a public health emergency, it is especially important to study psychological impact on the population in order to develop strategies to reduce symptoms during health crises (Wang et al., 2020).

Therefore, this study aimed to study the prevalence of people who have presented suicidal ideation during confinement in Spain, its relationship with anxiety-depressive symptomatology, and the role of social-emotional competencies in this relationship.

## Material and Methods

### Study Design

This study is part of a larger longitudinal survey, although the data below correspond to the first wave of cross-sectional data collection. This work is part of the Continuity Plan of the Miguel Hernández University of Elche to study and help the university community through a coordinated action plan in relation to possible psychological problems caused by the COVID-19 health crisis.

For this purpose, the “PsicorecurSOS COVID-19” online protocol was designed. This protocol aims to self-assess personal resources to cope with the psychological impact of the current health crisis. The survey was carried out using the online data collection platform DetectaWeb (Piqueras et al., 2017). The first page of the website provided information about the research team, interest of the study, objective, procedure, benefits and risks of participating, treatment of the information and confidentiality and contact information for any questions. Participants completed the survey online and, at the end, received a report with their personalized results along with recommendations and suggestions for the improvement of personal resources and the reduction of the health crisis’s impact.

The study was approved by the ethics committee of Miguel Hernández University (Reference: DPS.JPR.01.20). To encourage participation, the university, the Spanish Network of Healthy Universities (REUS), San Antonio de Murcia Catholic University, and the University of Alicante, disseminated information about the study through their websites and social networks, inviting the entire university community, their relatives and acquaintances to participate. Prior to completing the protocol, participants had to agree to their participation and provide informed consent. The data provided in this study correspond with the launch of the protocol (April 21, 2020) and the end of the state of alert in Spain (July 21, 2020).

### Participants

From an initial 1020 people who agreed to participate, we only included the data of those who provided informed consent to their data being used in the study, completed the entire protocol, and chose the option “male” or “female” in the question regarding sex. Therefore, this study analyzed data from 595 participants (58.30%), who were mostly women (72.44%).

The final sample mainly included working adults and students (68.90%) from Miguel Hernández University, whose ages ranged from 18 to 83 years (*M* = 37.18; *SD* = 13.20). Table 1 shows the sociodemographic characteristics of the final study sample.

**Table 1:** Sociodemographic Characteristics

	Total sample, *N* = 595 n (%)
Age	
17–18	7 (1.20)
19–25	140 (23.50)
26–40	206 (34.70)
50–60	214 (36.00)
60–90	28 (4.70)
Sex	
Male	164 (27.60)
Female	430 (72.40)
Marital Status	
Single	261 (43.80)
Married	190 (31.80)
Divorce	45 (7.60)
Live with a partner	100 (16.80)
Employment status	
Student	181 (30.40)
Active Worker	333 (56.00)
Inactive Worker	78 (13.10)

### Variables

#### Suicidal Ideation

Item 35 of the Anxiety and Depression Disorder Symptom Scale (ESTAD; Sandin et al., 2018) was used to evaluate participants’ suicidal ideation. The ESTAD is a self-report scale based on the DSM-5. It assesses seven dimensions of emotional disorder symptoms (agoraphobia, panic disorder, generalized anxiety disorder, social phobia, anxiety disorder, major depressive disorder, and obsessive-compulsive disorder). The item on suicide ideation asks whether a respondent had thought, “I have had thoughts of taking my life lately.” On a 5-point Likert scale ranging from 0 (never) to 4 (almost always), participants indicated how often this thought occurred since the beginning of the health crisis and confinement.

#### Anxious-Depressive Symptomatology

Through the four items of the Brief Patient Health Questionnaire (PHQ-4; Kroenke, Spitzer, & Williams, 2001), anxious-depressive symptomatology was evaluated. This instrument is an abbreviated version of the PHQ-9(Kroenke et al., 2001) and was used to determine the frequency during the last two weeks (from 0, not at all to 3, nearly every day) of the presence of these depression (PHQ-2) and anxiety (GAD-2) symptoms. Previous studies have shown this instrument to have high sensitivity, with cutoff points of three for each test (Muñoz-Navarro et al., 2017), and good psychometric properties (Cano-Vindel et al., 2018), with an alpha value of .83 and omega value of .90. This value is similar to that obtained in our study (α = .87).

#### Social-Emotional Skills

The Social Emotional Health Survey (SEHS; Furlong, You, Shishim, & Dowdy 2017) is used to study the presence of personal strengths or socio-emotional competencies and has two versions: one for university students (SEHS-HE), and one for the general population (SEHS-GP) by Piqueras et al. (2020). Both versions are identical, except for the questions referring to a university setting that in the SEHS-GP refer to a work setting (e.g., SEHS-HE: “I have a friend at my college who cares about me.” versus SEHS-GP: “I have a friend at my job who cares about me.”). This instrument is a multidimensional measure of Covitality, which refers to the coexistence of positive intrapersonal skills and interpersonal resources, the combination of which increases the probability a person will have positive results in their development and current psychological well-being. This is achieved by adding the results obtained in terms of belief in oneself (subscales: self-efficacy, persistence, self-awareness), belief in others (subscales: family support, institutional support, peer support), emotional competence (subscales: cognitive reevaluation, empathy, self-regulation), and committed life (subscales: gratitude, zest, optimism). This measure is obtained through 36 items with a response scale from 1, “very uncharacteristic of me,” to 6, “very characteristic” (for more information www.Covitalityucsb.info). Internal consistency analyses showed a Cronbach’s alpha value of .94, which was equal to that obtained by Furlong et al. (2017), who obtained reliability coefficients mainly adequate to excellent (ranging from .72 to .94). We also obtained a McDonald’s omega coefficient value of .96.

#### Fear of COVID-19

To evaluate fear of COVID-19 present in the participants due to the health crisis, the recently published Spanish version (Piqueras et al., 2020) of the Fear of COVID-19 Scale (FCV-19S; Ahorsu et al., 2020) was used. Through this instrument, participants had to indicate on a 5-point Likert scale (from 1 to 5) the degree of agreement or disagreement in relation to seven items that indicated fear of COVID-19, where the higher the score, the greater the fear of COVID-19. The FCV-19S has shown good internal consistency in previous studies, with a reported Cronbach alpha coefficient of 0.82 to 0.87 (Ahorsu et al., 2020). The Spanish adaptation has also shown evidence of reliability (Cronbach’s α = .86, McDonald’s ω = .86). These values are similar to those obtained in our study (α = .84, ω = .89).

### Statistical Analysis

SPSS software (Statistical Package for the Social Sciences, version 25) for Apple (IBM., 2016) was used to analyze the collected data.

**Fig. 1:**
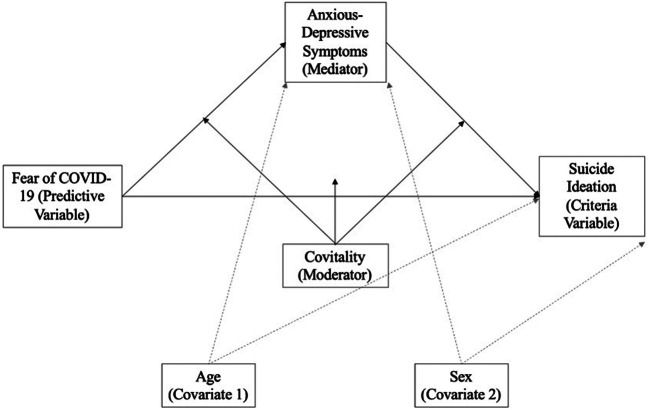
Conceptual and statistical diagram of Model 59

First, to determine the prevalence of suicidal ideation, fear of COVID-19, and anxiety-depressive symptoms, as well as the disposition of socio-emotional competencies or Covitality, descriptive analyses (*M* and *DT*) were carried out and frequencies were counted. Consequently, the mean scores of the variables (Student’s t test for independent samples and one-factor ANOVA) were compared according to sex and age, providing effect sizes using Cohen’s d (Cohen, 1988). Second, in relation to the scales, to assess internal consistency, Cronbach’s alpha and McDonald’s omega values were calculated (Cronbach, 1951; McDonald, 1999). An analysis of internal consistency was performed according to the reference values of George and Mallery (2003), in which a Cronbach’s alpha is considered questionable if between 0.61 and 0.70, acceptable if between 0.71 and 0.80, good if between 0.81 and 0.90, and excellent if above 0.90. Bivariate correlations were performed to examine the association between the study variables. Third, hierarchical multiple regression models were used to determine the predictive capacity of the different variables on suicidal ideation. Fourth, the computer tool SPSS PROCESS (Hayes, 2018) was used to carry out an analysis of conditional processes (model 59). Figure 1 shows the conceptual diagram of this analysis, where regression coefficients were estimated using the bootstrapping procedure (10,000 resamples) resulting in a 95% corrected bias and direct and indirect effect confidence intervals, where they are considered significant if there is no zero between the upper and lower confidence intervals (CI). A value less than 0.05 was considered significant

## Results

A total of 24.9% of the students had MDE-GAD at the 12-month follow-up. Table 2 shows the scores obtained by the participants. Regarding suicidal ideation, 12% responded affirmatively to the question about presence of ideas of suicide during the last 12 months (7.2% rarely, 2.9% quite often, 1.2% many times, and 0.7% almost always). For the presence of anxiety and depression, according to the cut-off scores established by Muñoz-Navarro et al. (2017), 24.0% and 19.3% exceeded the cut-off point for depression and anxiety, respectively. As shown in Table 2, there were statistically significant differences with medium effect sizes in the scores obtained for anxiety-depressive symptoms and fear of COVID-19, being higher in women.

**Table 2:** Descriptive statistics and sex differences

	Total (N = 595)			Men (*N* = 164)		Women (*N* = 431)		
	*Range of score*	*M*	*SD*	*M*	*SD*	*M*	*SD*	*d*
Fear of Covid	7–35	15.34	5.52	13.79	4.88	15.93	5.63	0.40***
Anxious-Depressive Symptoms	0–12	3.26	2.86	2.48	2.62	3.53	2.87	0.38***
Covitality	36–216	168.47	25.50	168.44	26.46	168.82	24.51	.01
Suicide Ideation	0–4	0.20	0.62	0.18	0.62	0.20	0.60	.03*

*Note*: *p < .05; ***p < .001; *M* = Mean; *SD* = Statistical Deviation; *d* = Cohen’s *d* effect size

Table 3 shows the correlations with the other variables. Age correlated with suicidal ideation, Covitality, and anxious-depressive symptoms.

**Table 3:** Bivariate correlations among study variables

	1	2	3	4	5
1. Age	1				
2. Fear of Covid	.49	1			
3. Anxious-Depressive Symptoms	−.33**	.32***	1		
4. Covitality	.21**	−.10***	−.47**	1	
5. Suicide Ideation	−.15*	.02	.35**	−.41**	1

*Note:* ^*^p <.05; ^**^p < .01; ^***^p <.001; Gender was coded 0 = male, 1 = female

As shown in Table 3, the correlations reflect differences according to age, being statistically significant and negative between anxiety-depressive symptomatology and suicidal ideation, and positive with Covitality. The highest correlations were between the total score of Covitality and the anxiety-depressive symptomatology and with suicidal ideation. The link of anxiety-depressive symptoms with suicidal ideation, age and fear of COVID-19 was also significant and positive. Therefore, due to the differences between the scores in the different groups, they were included as covariates in the moderate mediation model to control for effects.

To explore the possibility that fear of COVID-10 was related to suicidal ideation mediated by the presence of anxiety-depressive symptoms and moderated by Covitality, a moderate mediation model was constructed, which included age and sex as covariates.

**Fig. 2:**
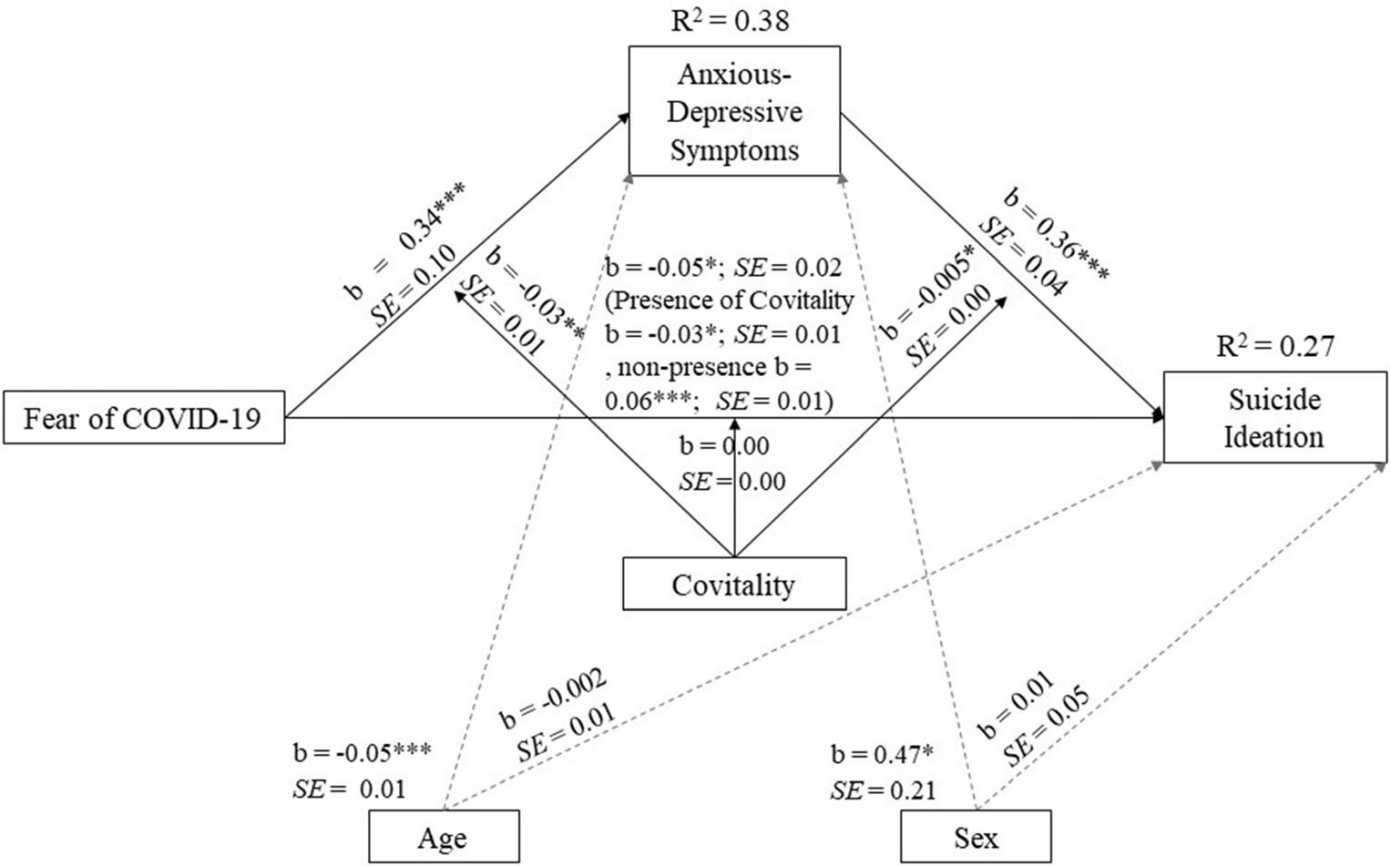
Moderate mediation model. *Note:* Statistical diagram of the mediation model for the influence of Fear of COVID-19 and its association with anxious-depressive symptoms as mediator and Covitality as moderator controlling age and sex. The values are represented with non-standardized regression coefficients. The value outside brackets represents the total effect and the value in brackets represents the direct effect of the bootstrapping analysis of Fear of Covid after the inclusion of the mediating, moderating and covariates variables

As can be seen in Fig. 2, this moderate mediation analysis showed that the participants who presented higher scores in fear of COVID-19, presence of anxiety-depressive symptoms, and lower scores in Covitality, presented a higher risk of reporting suicidal ideation, explaining 27% of the variance in suicidal ideation. In addition, the indirect effect of moderate mediation was significant (b = −.004, *SE* = .002 values of the lower limit (LLCI) and upper limit (ULCI) of the confidence interval of −.007 and − .0007 with the presence of Covitality and b = .01; *SE* = .003 with LLCI and ULVI of .004 and .017 absence of Covitality). Also, sex and age did not influence the overall outcome of the model.

## Discussion

### Main Findings

This study aimed to study the prevalence of suicidal ideation during confinement as well as to analyze the role of vitality in the presence of anxious-depressive symptoms and suicidal ideation in a Spanish sample.

First, in this study, the 12% responded affirmatively to the question about past suicidal ideation during the last 12 months. Concerning anxiety and depression symptoms, 24% and 19.3%, respectively, exceeded the clinical cutoff. Considering these data, it appears that the percentage of ideation during the period of confinement was higher than that reported in previous studies conducted with the general population before and during de pandemic. Before the pandemic, in the meta-analysis of Castillejos, Huertas, Martin, and Moreno (2020) on the prevalence of suicidal ideation in Europe, they found that, in 24 studies, the 12 months prevalence rate was 2.9%, and lifetime prevalence was 5.55%. In the study by Nock et al. (2008), with more than 85,000 adult participants, the reported lifetime prevalence was 9.2%. During the pandemic, in a Bangladeshi large-scale study with 10,067 participants, the 5.0% of participants reported suicidal ideation related to COVID-19 issues aftermath the pandemic (Mamun et al., 2020). However, in the study of Czeisler, et al. (2020) the percentage of 731 respondents (18–24 years old) who reported having seriously considered suicide in the 30 days before completing the survey was higher (25.5%) There was therefore an increase in suicidal ideation during the confinement period. Concerning anxiety and depression, a survey was conducted in Spain in 2017 in which more than 29,195 non-institutionalized people over the age of 15 years participated (Spanish National Health Survey: ENSE, 2017). In the ENSE, 4% of men and 9.2% of women self-reported anxiety, and 4.3% of men and 9.1% of women reported depression. These data were lower than those in our study. However, similar results have also been found in previous studies regarding increase in the presence of this symptomatology. In Spain, both in the study by Ozamiz-Etxebarría, Dosil-Santamaria, Picaza-Gorrochategui, & Idoiaga-Mondragon(2020) with 976 adults and the study by González-Sanguino et al., (2020) with 3480, during March, 11.84% and 18.7% of their participants presented symptoms of depression and 24.62% and 21.60% anxiety, respectively. Similar percentages were also reflected in a study with a Chinese population, with 16.5% of participants presenting depressive symptoms (moderate to severe), and 28.8% anxiety during the initial stage of the 2019 coronavirus disease. (Wang et al., 2020). Regarding the Covitality score, in a previous study with university students aged 17 to 25 years, the mean score was 163.35 (*SD* = 20.49; Soto-Sanz et al., 2018), similar to that found in this age group in this study (*M* = 162.72, *SD* = 24.29). There is thus not enough information to determine whether the score found during confinement due to COVID-19 and in the different age groups, is different or not.

Second, there were differences between sex and age. Women in this study scored higher than men on anxious-depressive symptoms and fear of COVID-19, with medium effect sizes. As for age, the correlation reflected that being younger was correlated with higher scores in anxious-depressive symptoms and suicidal ideation, and older with higher scores in Covitality. In relation to anxious-depressive symptoms, being older was significantly negatively related with depression and anxiety, as in the study by Gonzalez-Sanguino et al. (2020), with women exhibiting higher scores in anxious-depressive symptoms both in their study and ours. Regarding fear of COVID-19, previous studies have highlighted the difference between sexes, with fear being significantly higher in women (Bakioglu, Korkmaz, & Ercan, 2020). Additionally, in the study by Castillejos et al. (2020), an association was found between lower age and higher suicidal ideation scores.

Third, the moderate mediation analysis revealed an explained variance of 27%. Participants with higher scores in fear of COVID-19, presence of anxious-depressive symptoms, and low Covitality, were at higher risk of reporting suicidal ideation. In relation to these findings, no studies were found where the COVID-19 fear score and its relation to suicidal ideation were analyzed through anxiety-depressive symptoms and social-emotional competencies in the general population. However, it has been found that anxious-depressive symptoms mediate between COVID-19 fear and life satisfaction (Satici, Gocet-Tekin, Deniz, & Satici, 2020a) and that positive factors may protect against suicidal ideation (Chang et al., 2017; Sánchez-Alvarez et al., 2020). As regards the relationship between anxious-depressive symptoms and suicide ideation, previous studies have shown this to be a positive relationship (Blasco et al., 2019; Soto-Sanz et al., 2019). Fear of COVID-19 has previously been associated with depression and anxiety symptoms as a statistically significant variable (Fitzpatrick et al., 2020; Harper et al., 2020; Satici et al., 2020a).

### Limitations

Several limitations of this study should be acknowledged. First, the cross-sectional design did not provide evidence of a causal relationship between fear of COVID-19, anxious-depressive symptoms, Covitality, and suicidal ideation. Our findings, along with previous research, suggest a mechanism through which these factors may be related; longitudinal research is needed to determine whether the direction of the correlations may differ from what is assumed in our theoretical model, although there is empirical evidence that fear of COVID-19 precedes changes in anxious-depressive symptoms, and this influence of suicide ideation, experimental and longitudinal perspectives would help to clarify causal pathways. Second, although the use of self-report measures has shown good reliability, shared method variance may have inflated relationships found between instruments. Hence, future studies would benefit from using other methods to generalize our findings (e.g., multiple informants) and test other results related to suicide risk. Relatedly, other questions about suicide risk are needed to complement suicidal ideation items in order to test more complex models of suicide risk. Another limitation is that the survey only required the identification of participants as male/female, so gender and/or sexual identity data were not available. As a result, possible differences in the impact of COVID-19 on the population depending on their gender or sexual identity was not included. In addition, gender has been identified as a relevant variable in relation with mental health differences (Rosenfield & Mouzon, 2013) as well as sex identity (Nam, Jun, Fedina, Shah, & DeVylder, 2019).

It would be interesting to examine the longitudinal effects of Covitality on suicidal ideation and behaviors, as this would improve our understanding of the temporal dynamics of the associations between factors relevant to bullying. Despite these limitations, our findings provide an initial step towards a better understanding of the indirect effect of anxious-depressive symptoms and Covitality via its association with fear of COVID-19 and suicidal ideation and suggest that Covitality may be an appropriate target for therapeutic programs in suicide prevention efforts among people with fear of COVID-19 and anxious-depressive symptoms.

Finally, in this study, the differences between the different age and sex groups did not affect the model. However, the sample size in the different groups was too different to be able to adequately study these possible differences. More research on the detection of risk groups is needed, following the study carried out by Pakpour and Griffiths (2020) that emphasized the importance of determining risk groups based on socio-demographic variables in order to develop prevention programs to help overcome fear of COVID-19.

## Conclusion

The current study provides important contributions to the possible causes of the current mental health crisis and the protective role of social-emotional competencies, as it suggests that people without social-emotional competencies who present fear of COVID-19 and anxiety-depressive symptoms are more at risk of suicidal ideation. Covitality moderates the impact of COVID-19 fear on suicidal ideation through anxiety-depressive symptomatology. This confirms the importance of social-emotional skills to protect against the effects of fear of COVID-19.

Suicide is a multi-causal phenomenon and it is important not to ignore the influence of the health crisis on the different factors. The consequences of the pandemic may lead to a confluence of risk factors resulting in increased rates of suicide and long-term suicide attempts (Reger, Stanley, & Joiner, 2020). Therefore, beyond anxiety and depression, many of the risk factors associated with suicide have worsened during the COVID-19 crisis by increasing the risk or likelihood of occurrence of suicidal behavior or ideation. For example, isolation, coping with death and economic hardship (Druss, 2020), low socioeconomic status, belonging to low-income countries, and unemployment have increased rates and risk (Gunell and Chang, 2016; International Labor Organization, 2020).

Our study results suggest that, although further longitudinal studies are needed to confirm our results, individuals with belief in self (self-awareness, self-efficacy, and persistence), belief in others (academic or work support, family coherence, and peer support), have emotional competence (emotion regulation, empathy, and self-control), and engaged living (optimism, zest, and gratitude) are more protected against the impact of the fear of COVID-19, while anxious-depressive symptoms may cause suicidal ideation.

It is important to note that, in this study, the reported effects are small and based in a non-representative convenience sample. Although statistically significant results are provided, this statistical significance comes from a combination of the effect in the sample being large enough but the data may be of better quality. One requirement is that participation among men and women would have been equal for the data to be of adequate quality for our estimates of the effect to have sufficient precision to understand these values with greater confidence.

However, we consider the subject of this study to be suicide. It is a topic of great relevance and concern; therefore, we consider it important to consider these results paying attention to the limitations. This implies that, in the face of a crisis of this type, the population must be prepared psychologically and must strengthen their socio-emotional competencies. This suggests prevention and management of effective socio-health measures because it is possible to truly operationalize primary prevention in mental health (Wolf, 2012). Entities such as the European Observatory on Health Systems and Policies, the Organization for Economic Co-operation and Development, and the WHO Regional Office for Europe have collected data indicating that influencing the risk behavior for different diseases, including mental health disorders, is an efficient use of government resources, and that government policies can have a high impact on risk behavior for mental health disorders (McDaid, Sassi, & Merkur, 2015).

Unfortunately, the high risk of suicide may be the result of an undiagnosed mental disorder or long periods without access to available treatments (de Girolamo et al., 2012). Therefore, early detection of emotional symptoms can be a good measure to prevent suicide—specifically, by using online screening measures for lower costs and faster detection.

To this end, preventive actions should be designed at various levels. First, at a universal level, with two mental health education-related measures. The first measure should focus on raising public awareness of the detection of changes in mental state as a result of COVID-19. This would involve educating the population on the need to self-explore for possible psychological changes (just as self-exploration is encouraged in breast cancer prevention). The second measure, which would focus on carrying out mental health and well-being promotion campaigns, would emphasize social-emotional skills training. Second, there should be prevention, online detection, and intervention in people whose mental health is affected by the health crisis. In short, growing evidence suggests that preventive interventions in psychiatry are feasible, safe, and cost-effective. Universal, indicated, and targeted prevention strategies could be effective in improving psychological well-being or preventing mental disorders (Arango et al., 2018).

In sum, in this situation of unprecedented confinement for the Spanish population, it is very important to attend to the psychological factors of the population. It is also necessary to continue studying the effects that the COVID-19 crisis will have on people’s health. To that end, this study aimed to help generate social and health treatment initiatives to prevent and alleviate the psychosocial effects of the pandemic. Thus, this work can provide an interesting starting point for further research on the importance of socio-emotional skills—namely, Covitality.

## Data Availability

The datasets generated during and/or analysed during the current study are available from the corresponding author on reasonable request.

## References

[CR1] Ahorsu, D. K., Lin, C. Y., Imani, V., Saffari, M., Griffiths, M. D., & Pakpour, A. H. (2020). The fear of COVID-19 scale: Development and initial validation. *International Journal of Mental Health and Addiction*, *1*–*9*. 10.1007/s11469-020-00270-8. 10.1007/s11469-020-00270-8 PMC7100496 32226353

[CR2] Arango C, Díaz-Caneja CM, McGorry PD, Rapoport J, Sommer IE, Vorstman JA, McDaid D, Marín O, Serrano-Drozdowskyj E, Freedman R, Carpenter W (2018). Preventive strategies for mental health. The Lancet Psychiatry.

[CR3] Bakioğlu, F., Korkmaz, O., & Ercan, H. (2020). Fear of COVID-19 and positivity: Mediating role of intolerance of uncertainty, depression, anxiety, and stress. *International journal of mental health and addiction*, 1. 10.1007/s11469-020-00331-y. 10.1007/s11469-020-00331-y PMC7255700 32837421

[CR4] Bar-On R (1997). The emotional quotient inventory (EQ-i): Technical manual.

[CR5] Blasco MJ, Vilagut G, Almenara J, Roca M, Piqueras JA, Gabilondo A, Echeburúa E (2019). Suicidal thoughts and behaviors: Prevalence and association with distal and proximal factors in Spanish university students. Suicide and Life-Threatening Behavior.

[CR6] Caballero-Domínguez, C. C., Jiménez-Villamizar, M. P., & Campo-Arias, A. (2020). Suicide risk during the lockdown due to coronavirus disease (COVID-19) in Colombia. *Death Studies*, 1–6. 10.1080/07481187.2020.1784312. 10.1080/07481187.2020.1784312 32589519

[CR7] Cano-Vindel A, Muñoz-Navarro R, Medrano LA, Ruiz-Rodríguez P, González-Blanch C, Gómez-Castillo MD, PsicAP Research Group (2018). A computerized version of the patient health Questionnaire-4 as an ultra-brief screening tool to detect emotional disorders in primary care. Journal of Affective Disorders.

[CR8] Castillejos, M. C., Huertas, P., Martín, P., & Moreno Küstner, B. (2020). Prevalence of suicidality in the European general population: A systematic review and Meta-analysis. *Archives of Suicide Research*, 1–19. 10.1080/13811118.2020.1765928. 10.1080/13811118.2020.1765928 32620069

[CR9] Cohen J (1988). Statistical power analysis for the behavioral sciences.

[CR10] Cronbach LJ (1951). Coefficient alpha and the internal structure of tests. Psychometrika.

[CR11] Czeisler, M. É., Lane, R. I., Petrosky, E., Wiley, J. F., Christensen, A., Njai, R., ... & Rajaratnam, S. M. (2020). Mental health, substance use, and suicidal ideation during the COVID-19pandemic—United States, June 24–30, 2020. *Morbidity and Mortality Weekly Report*, *69*(32), 1049. 10.15585/mmwr.mm6932a1 10.15585/mmwr.mm6932a1 PMC7440121 32790653

[CR12] de Girolamo, G., Dagani, J., Purcell, R., Cocchi, A. y McGorry, P. D. (2012). Age of onset of mental disorders and use of mental health services: Needs, opportunities and obstacles. Epidemiology and Psychiatric Sciences*,* 21, 47–57. 10.1017/S2045796011000746. 10.1017/s2045796011000746 22670412

[CR13] de Hoog N, Stroebe W, de Wit JB (2008). The processing of fear-arousing communications: How biased processing leads to persuasion. Social Influence.

[CR14] Domínguez-García E, Fernández-Berrocal P (2018). The association between emotional intelligence and suicidal behavior: A systematic review. Frontiers in Psychology.

[CR15] Druss BG (2020). Addressing the COVID-19 pandemic in populations with serious mental illness. JAMA Psychiatry..

[CR16] Dsouza, D. D., Quadros, S., Hyderabadwala, Z. J., & Mamun, M. A. (2020). Aggregated COVID-19 suicide incidences in India: Fear of COVID-19 infection is the prominent causative factor. *Psychiatry research*, 113145 doi:10.31234/osf.io/7xa4b. 10.1016/j.psychres.2020.113145 PMC7832713 32544650

[CR17] Fitzpatrick KM, Harris C, Drawve G (2020). Fear of COVID-19 and the mental health consequences in America. Psychological Trauma: Theory, Research, Practice, and Policy..

[CR18] Furlong MJ, You S, Renshaw T, O’Malley MD, Rebelez J (2013). Preliminary development of the positive experiences at school scale for elementary school children. Child Indicators Research.

[CR19] Furlong MJ, You S, Shishim M, Dowdy E (2017). Development and validation of the social emotional health survey–higher education version. Applied Research in Quality of Life.

[CR20] George, D., & Mallery, M. (2003). Using SPSS for windows step by step: A simple guide and reference*.*

[CR21] Gili M, Castellví P, Vives M, de la Torre-Luque A, Almenara J, Blasco MJ (2019). Mental disorders as risk factors for suicidal behavior in young people: A meta-analysis and systematic review of longitudinal studies. Journal of Affective Disorders.

[CR22] Gilmour, H. L. (2016). *Threshold and subthreshold generalized anxiety disorder (GAD) and suicide ideation* (pp. 13–21). Statistics Canada. 27849314

[CR23] Goleman D (1995). Emotional intelligence: Why it can matter more than IQ bantam books.

[CR24] González-Sanguino C, Ausín B, Castellanos M, Saiz J, López-Gómez A, Ugidos C, Muñoz M (2020). Mental health consequences during the initial stage of the 2020 coronavirus pandemic (COVID-19) in Spain. Brain, Behavior, and Immunity..

[CR25] Goyal K, Chauhan P, Chhikara K, Gupta P, Singh MP (2020). Fear of COVID 2019: First suicidal case in India!. Asian Journal of Psychiatry.

[CR26] Gunnell D, Chang S, O’Connor RC, Pirkis J (2016). Economic recession, unemployment, and suicide. The international handbook of suicide prevention.

[CR27] Harper, C. A., Satchell, L. P., Fido, D., & Latzman, R. D. (2020). Functional fear predicts public health compliance in the COVID-19 pandemic. *International Journal of Mental Health Addiction.*10.1007/s11469-020-00281-5. 10.1007/s11469-020-00281-5 PMC7185265 32346359

[CR28] Hayes, A. F. (2018). Introduction to mediation, moderation, and conditional process analysis second edition: A regression-based approach. New York, NY*:* Ebook The Guilford Press. Google Scholar.

[CR29] International Labour Organization (ILO), 2020. Available from https://www.ilo.org/global/about-the-ilo/newsroom/news/WCMS_739961/lang%2D%2Den/index.htm

[CR30] Korkmaz S, Keleş DD, Kazgan A, Baykara S, Gürok MG, Demir CF, Atmaca M (2020). Emotional intelligence and problem solving skills in individuals who attempted suicide. Journal of Clinical Neuroscience..

[CR31] Kroenke K, Spitzer RL, Williams JB (2001). The PHQ-9: Validity of a brief depression severity measure. Journal of General Internal Medicine.

[CR32] Li S, Wang Y, Xue J, Zhao N, Zhu T (2020). The impact of COVID-19 epidemic declaration on psychological consequences: A study on active Weibo users. International Journal of Environmental Research and Public Health.

[CR33] Mamun MA, Griffiths MD (2020). First COVID-19 suicide case in Bangladesh due to fear of COVID-19 and xenophobia: Possible suicide prevention strategies. Asian Journal of Psychiatry.

[CR34] Mamun MA, Sakib N, Gozal D, Bhuiyan AI, Hossain S, Bodrud-Doza M, al Mamun F, Hosen I, Safiq MB, Abdullah AH, Sarker MA, Rayhan I, Sikder MT, Muhit M, Lin CY, Griffiths MD, Pakpour AH (2020). The COVID-19 pandemic and serious psychological consequences in Bangladesh: A population-based nationwide study. Journal of Affective Disorders.

[CR35] Mayer JD, Salovey P (1997). What is emotional intelligence. Emotional development and emotional intelligence: Educational implications.

[CR36] McDaid D, Sassi F, Merkur S (2015). Promoting health, preventing disease the economic case.

[CR37] McDonald RP (1999). Test theory: A unified approach.

[CR38] Ministerio de Sanidad, Consumo y Bienestar Social del Gobierno de España (2020). Situación actual del COVID-19 en España. Retrieved June 1, 2020 from https://cnecovid.isciii.es/covid19/

[CR39] Mortensen CR, Becker DV, Ackerman JM, Neuberg SL, Kenrick DT (2010). Infection breeds reticence: The effects of disease salience on self-perceptions of personality and behavioral avoidance tendencies. Psychological Science.

[CR40] Muñoz-Navarro R, Cano-Vindel A, Ruiz-Rodríguez P, Medrano LA, González-Blanch C, Moriana JA (2017). Modelo jerárquico de diagnóstico y derivación de los trastornos mentales comunes en centros de atención primaria. Una propuesta a partir del ensayo clínico PsicAP. Ansiedad y estrés.

[CR41] Nam B, Jun HJ, Fedina L, Shah R, DeVylder JE (2019). Sexual orientation and mental health among adults in four US cities. Psychiatry Research.

[CR42] Ozamiz-Etxebarria N, Dosil-Santamaria M, Picaza-Gorrochategui M, Idoiaga-Mondragon N (2020). Niveles de estrés, ansiedad y depresión en la primera fase del brote del COVID-19 en una muestra recogida en el norte de España. Cadernos de Saúde Pública.

[CR43] Pakpour, A. H., & Griffiths, M. D. (2020). The fear of COVID-19 and its role in preventive behaviors. *Journal of Concurrent Disorders*. ISSN 2562-7546.

[CR44] Petrides KV, Pita R, Kokkinaki F (2007). The location of trait emotional intelligence in personality factor space. British Journal of Psychology.

[CR45] Piqueras JA, García-Olcina M, Rivera-Riquelme M, Rodríguez-Jiménez T, Martínez-González AE, Cuijpers P (2017). DetectaWeb project: Web-based detection of mental health continuum in children and adolescents. BMJ Open.

[CR46] Piqueras, J. A., Gomez-Gomez, M., Marzo, J. C., Gomez-Mir, P., Falcó, R., Valenzuela, B., Rivera-Riquelme, M., & PSICORECUR-SOS COVID-19 study group. (2020). Validation of the Spanish version of the fear of COVID-19 scale: Its association with acute stress and coping. *International Journal of Mental Health and Addiction.* 10.1007/s11469-021-00615-x PMC8500468 34642579

[CR47] Renshaw, T. L., Furlong, M. J., Dowdy, E., Rebelez, J., Smith, D. C., O’Malley, M. D., ... Strøm, I. F. (2014). Covitality: A synergistic conception of adolescents’ mental health. In M. J. Furlong, R. Gilman, & E. S. Huebner (Eds.), *Handbook of positive psychology in the schools* (2nd ed., pp. 12–32). Routledge.

[CR48] Rosenfield S, Mouzon D, Aneshensel CS, Phelan JC, Bierman A (2013). Gender and mental health. Handbook of the sociology of mental health.

[CR49] Sandín B, Valiente RM, Pineda D, García-Escalera J, Chorot P (2018). Escala de Síntomas de los Trastornos de Ansiedad y Depresión (ESTAD): Datos preliminares sobre su estructura factorial y sus propiedades psicométricas. Revista de Psicopatología y Psicología Clínica.

[CR50] Sareen J, Cox BJ, Afifi TO, de Graaf R, Asmundson GJ, Ten Have M, Stein MB (2005). Anxiety disorders and risk for suicidal ideation and suicide attempts: A population-based longitudinal study of adults. Archives of General Psychiatry.

[CR51] Satici, B., Gocet-Tekin, E., Deniz, M. E., & Satici, S. A. (2020a). Adaptation of the fear of COVID-19 scale: Its association with psychological distress and life satisfaction in Turkey. *International Journal of Mental Health Addiction.*10.1007/s11469-020-00294-0. 10.1007/s11469-020-00294-0 PMC7207987 32395095

[CR52] Satici, B., Saricali, M., Satici, S. A., & Griffiths, M. D. (2020b). Intolerance of uncertainty and mental wellbeing: Serial mediation by rumination and fear of COVID-19. *International Journal of Mental Health and Addiction.*10.1007/s11469-020-00305-0. 10.1007/s11469-020-00305-0 PMC7228430 32427165

[CR53] Schaller M, Murray DR (2008). Pathogens, personality, and culture: Disease prevalence predicts worldwide variability in sociosexuality, extraversion, and openness to experience. Journal of Personality and Social Psychology.

[CR54] Soto-Sanz, V., Mira-López, F., Marzo, J.C., Piqueras, J.A., & Covitality team. (2018). Sintomatología Internalizante y Externalizante y la Covitalidad como factor protector en estudiantes universitarios. 4th International Congress of Clinical and Health Psychology on Children and Adolescents.

[CR55] Soto-Sanz V, Castellví P, Piqueras JA, Rodríguez-Marín J, Rodríguez-Jiménez T, Miranda-Mendizábal A (2019). Internalizing and externalizing symptoms and suicidal behaviour in young people: A systematic review and meta-analysis of longitudinal studies. Acta Psychiatrica Scandinavica.

[CR56] Wang C, Pan R, Wan X, Tan Y, Xu L, Ho CS, Ho RC (2020). Immediate psychological responses and associated factors during the initial stage of the 2019 coronavirus disease (COVID-19) epidemic among the general population in China. International Journal of Environmental Research and Public Health.

[CR57] Weare K (2015). What works in promoting social and emotional well-being and responding to mental health problems in schools.

[CR58] Wolf H, Bährer-Kohler S (2012). Self- management and mental health. 2012.

[CR59] World Health Organization (WHO) (2020). Coronavirus disease (COVID-19) outbreak situation. Retrieved June 1, 2020 from https://www.who.int/emergencies/diseases/novel-coronavirus-2019

